# Clinical benefits of oral capecitabine over intravenous 5-fluorouracyl regimen in case of neoadjuvant chemoradiotherapy followed by surgery for locally advanced rectal cancer

**DOI:** 10.3389/pore.2022.1610722

**Published:** 2022-12-08

**Authors:** Attila Paszt, Aurel Ottlakan, Szabolcs Abraham, Zsolt Simonka, Marton Vas, Aniko Maraz, Zoltan Szepes, Laszlo Tiszlavicz, Tibor Nyari, Judit Olah, Gyorgy Lazar

**Affiliations:** ^1^ Department of Surgery, University of Szeged, Szeged, Hungary; ^2^ Department of Oncotherapy, University of Szeged, Szeged, Hungary; ^3^ 1st Department of Internal Medicine, University of Szeged, Szeged, Hungary; ^4^ Department of Pathology, University of Szeged, Szeged, Hungary; ^5^ Department of Medical Physics and Informatics, University of Szeged, Szeged, Hungary

**Keywords:** surgery, chemoradiotherapy, neoadjuvant treatment, capecitabine, advanced rectal cancer

## Abstract

**Background:** During the last decade, one of the most important treatment options for locally advanced, potencially resectable rectal tumours was neoadjuvant chemoradiotherapy (CRT) followed by surgery.

**Methods:** Effects of the neoadjuvant treatment on surgical outcomes were retrospectively analysed in 185 patients with stage T2–T4 and N0–2, resectable rectal tumour among two patient groups defined by radiosensitizer agents. Group 1 (*n* = 94) involved radiotherapy (RT) with 50.4 Gy total dose (25 × 1.8 Gy + 3 × 1.8 Gy tumour bed boost), and intravenous 5-fluorouracil (5-FU) (350 mg/m^2^) with leucovorin (20 mg/m^2^) on the 1–5 and 21–25 days, while Group 2 (*n* = 91) RT and orally administrated capecitabine (daily 2 × 825 mg/m^2^) on RT days. Surgery was carried out after 8–10 weeks. Side effects, perioperative complications, type of surgery, number of removed regional lymph nodes, resection margins and tumour regression grade (TRG) were analysed.

**Results:** More favourable side effects were observed in Group 2. Despite the same rate of diarrhoea (Group 1 vs. Group 2: 54.3% vs. 56.0%), Grade 2–3 diarrhoea ratio was lower (*p* = 0.0352) after capecitabine (Group 2). Weight loss occurred in 17.0% and 2.2% (*p* = 0.00067), while nausea and vomiting was described in 38.3% and 15.4% (*p* = 0.00045) with 5-FU treatment and capecitabine respectively. Anaemia was observed in 33.0% and 22.0% (*p* = 0.0941). Complete tumour regression occurred in 25.3% after oral- and 13.8% after intravenous treatment (*p* = 0.049). Ratio of sphincter preservation was higher with laparoscopy than open surgery (72.3% vs. 39.7%) (*p* = 0.00001).

**Conclusion:** The study confirms advantages of neoadjuvant chemoradiotherapy with oral capecitabine for rectal tumours, such as more favourable side effect profile and overall clinical outcome, with increased rate of complete tumour regression.

## Introduction

Neoadjuvant oncological treatment has been a valuable part of treatment armament for over 2 decades. First development of treatment protocols started in the 1990s. Early studies on neoadjuvant radiotherapy of rectal tumours reported relatively low doses (40 Gy/2 Gy fractions), which resulted in improved disease-free survival compared to results of surgery alone [1,2].

Early treatment protocols were specifically developed for advanced stage, inoperable solid tumours after moderate oncological response. Treatments with modified indications were introduced later. The aim of these cases, was not only to achieve operability, but also to preserve organs, with improved locoregional control and improved survival [3].

Besides improvement in surgical technique, effectiveness was also enhanced with increased doses of radiation, techniques and chemotherapeutic radiation sensitisation using 5-fluorouracil (5-FU) [4] and hypofractionation, using single, increased, short course radiation fraction doses [5].

In case of rectal tumours, pre-treatment showed promising results in case of T3–4 and N0–2 stages and in T2 tumours near the sphincter [6].

Compared to postoperative chemoradiotherapy, preoperative application improved local control, however it did not improve overall survival. On the other hand, after a 5-year follow-up the surgery only approach, with short-course, high dose preoperative radiotherapy showed reduced local recurrence rates and improved survival in patients with resectable rectal tumours [7].

Since associated with less toxicity and a higher rate of sphincter preservation, neoadjuvant chemoradiotherapy has become widely accepted [8].

Despite its many advantages, intravenous chemotherapy has a markedly adverse impact on patient quality of life, and is associated with numerous side effects. The use of an effective oral drug has long been a subject of interest.

Since 5-FU is poorly absorbed orally and immediately catabolised by dihydropyrimidine dehydrogenase, capecitabine served as a potential alternative due to its simple oral dosing, excellent absorption, and role as a tumour-selective prodrug (fluoropyrimidine carbamate). Via a three-step enzymatic reaction it is converted intracellularly into an active metabolite. The fact that the last conversion is assisted by thymidine phosphorylase—the concentration of which is considerably higher in the tumour compared to healthy tissue—grants tumour selectivity during treatment [9].

While 5-FU can only be administered intravenously, capecitabine is an oral drug, and can therefore be used much easier in a well-controlled manner. It is also easy and safe for patients to handle administration at home. Preoperative chemoradiotherapy combined with oral capecitabine is safe and well-tolerated [10], results in potential downstaging, and may increase rates of sphincter preserving surgery [11]. The number of well-known and frequently occurring dose related complications (catheter-related infections, pneumothorax, thrombosis or bleeding) are also significantly lower [12].

New treatment direction involves a risk-adapted preoperative therapy performed in accordance with initial stage determined using increasingly accurate diagnostic options, such as multiparameter rectal magnetic resonance imaging (MRI). Up-to-date treatment may not only include chemoradiation but also short-course radiotherapy and in locally advanced cases (before or after radiotherapy), integrated preoperative oxaliplatin-capecitabine chemotherapy [13].

### Objective

Two approaches have been used before the latest expansion of total neoadjuvant therapy (TNT) in the neoadjuvant chemoradiation of locally advanced rectal tumours treated at our department between 2012–2014 and 2014–2017.

The component of chemotherapy used during concomitant neoadjuvant therapy has been changed in the current study. Instead of intravenous 5-FU, patients received oral capecitabine with unchanged protocol for radiotherapy.

We analysed the side effects of the two treatment protocols and postoperative complications after changed regimen with evaluation of the ratio for resection/extirpation surgery. Changes in the pathological regression of tumours, lymph node status and size of resection margins after different treatments were also evaluated.

## Materials and methods

Treatment outcomes of patients with stage T2–4 and N0–2 rectal tumours receiving neoadjuvant oncological treatment at the University of Szeged between 14 February 2012 and 28 March 2017 were retrospectively evaluated. The study was approved by the Regional and Institutional Review Board of Human Investigations at the University of Szeged, Hungary, approval number: 117/2020-SZTE.

### Patient groups

The study included two groups, the first one included patients who received long course radiotherapy potentiated with an intravenously administered chemotherapeutic agent (5-FU, *n* = 94), while the second group consisted of patients treated with unchanged radiotherapy combined with an oral drug (capecitabine, *n* = 91) (Table 1.).

**TABLE 1:** Summarizing table.

Type of neoadjuvant therapy	5 FU (inravenous group, *n* = 94)			Capecitabine (oral group, *n* = 91)				*p*-value
Age (with SD)	62.8 ± 11.2 years			63.1 ± 9.0 years				*p* = 0.858^a^
Sex (no.)	Female:33	Male:61		Female:27		Male:64		*p* = 0.43^b^
BMI (with SD)	26.8 ± 5.1			26.9 ± 5.3				*p* = 0.905^a^
Type of surgery (no.)	Open:50	Laparoscopic:44		Open:23		Laparoscopic: 68		*p* = 0.0001^b^
Type of open surgery (no.)	Resection: 19	Extirpation: 31		Resection: 10		Extirpation: 13		*p* = 0.657^b^
Type of laparoscopic surgery (no.)	Resection: 33	Extirpation: 11		Resection: 48		Extirpation: 20		*p* = 0.61^b^
Protective ileostomy (no.)	36			49				*p* = 0.0054^b^
Distal resection margin distance at open surgery (with SD)	17 ± 12 mm			14 ± 8 mm				*p* = 0.296^c^
Distal resection margin distance at laparoscopic surgery (with SD)	28 ± 15 mm			23 ± 13 mm				*p* = 0.892^c^
Circumferential resection margin distance at open surgery (with SD)	9 ± 8 mm			10 ± 7 mm				*p* = 0.545^c^
Circumferential resection margin distance at laparoscopic surgery (with SD)	13 ± 9 mm			13 ± 10 mm				*p* = 0.95^c^
Initial T stage (patient)	T_2_:1	T_3_: 80	T_4_: 13	T_x_: 1	T_3_: 78		T_4_: 12	*p* = 0.6129^b^
Initial N stage (patient)	N_0_: 35	N_1_: 51	N_2_: 8	N_x_: 1	N_0_: 40	N_1_: 43	N_2_: 7	*p* = 0.6082^b^
Initial M stage (patient)	M_x_: 3	M_0_: 78	M_1_: 13	M_x_: 8	M_0_: 80	M_1_: 3		*p* = 0.0142^b^

^a^ Two-sample student t-test.

^b^ Chi-squared test.

^c^ One way ANOVA test.

### Patient investigation and preoperative staging

Patients were subjected to colonoscopy, histological sample collection and staging before oncological treatment. Tumour staging included abdominal, and chest CT (computed tomography) or MRI and/or endosonography [14]. After oncological treatment, restaging (CT or MRI) [15,16] was performed. Prior to surgery, preoperative work-up was carried out (laboratory tests, coagulation parameters, blood type test, chest X-ray, cardiac assessment and consultation with an anaesthetist).

### Decision by the tumour board

Decision on neoadjuvant treatment was made in each case by the multidisciplinary (oncology) tumour board. Provided that the patient accepted decision on pre-treatment, initiation of neoadjuvant CRT could be carried out. Patients with metastatic or irresectable disease were excluded from this study.

### Neoadjuvant radiotherapy

Patient positioning, target volumes and planning: Topometric CT was performed in most cases in prone position using belly board, with individual immobilization system and thermoplastic mask fixation. Polystyrene wedge was placed between the buttocks. Topometric CT was performed on a Somatom Emotion 6 CT simulator (Siemens, Erlangen, Germany). Target volumes (rectum, rectal cancer, perirectal and regional pelvic lymph nodes) and organs at risk were delineated by an experienced radiologist in all cases. For treatment planning Eclipse planning system was used (Varian Oncology Systems). Radiation was delivered using a 3D conformal four-field box technique. Planned radiation dose was 25 times 1.8 Gy daily, continued with 3 fractions as the tumour bed boost. RT began on the first day of chemotherapy and was administered five times per week.

### Neoadjuvant chemotherapy

Neoadjuvant radiotherapy was completed by neoadjuvant chemotherapy, during which a radiotherapy-sensitising chemotherapeutic drug was administered. During a previously accepted treatment protocol, antimetabolite 5-fluorouracil was completed with leucovorin, which raises the inhibition of thymidylate synthesis, to increase therapeutic effect [17,18].

**FIGURE 1:**
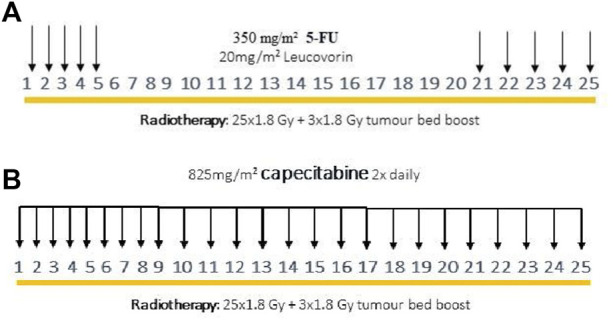
Previous neoadjuvant oncological treatment scheme with intravenous 5-FU and leucovorin **(A)**, modified neoadjuvant oncological treatment scheme with capecitabine **(B)**.

Figure 1 shows a schematic outline of the two types of neoadjuvant treatment administered at our department. Radiotherapy always consisted of previously described CRT, with a total dose of 50.4 Gy, although there were two types of additional chemotherapies. Whereas 5-FU and leucovorin were both previously added to RT, as of 2014, RT has been combined with the capecitabine-containing drug to potentiate radiotherapy [19].

Neoadjuvant CRT potentiated with 5-FU: During previous oncological protocol, 5-FU (350 mg/m^2^) and leucovorin (20 mg/m^2^) were given intravenously on the first and last 5 days of radiotherapy (Figure 1A).

Neoadjuvant CRT potentiated with capecitabine: During the modified oncological protocol, oral capecitabine (825 mg/m^2^) was administered on each day of radiotherapy (Figure 1B).

### Surgical treatment

Surgeries were performed by 14 experienced colorectal surgeons (ten in the previous period and 13 in the new period). Both open and laparoscopic techniques were performed by each surgeon. Surgical approach was based on the surgeon’s individual preference.

Open surgeries involved complete total mesorectal excision (TME) *via* lower midline laparotomy. The resection entailed high ligation of the inferior mesenteric artery and vein, and the anastomosis was created using double stapler technique in each case. In case of lower-third tumours, a perineal, rectorectal drain was placed; otherwise, abdominal drainage was carried out. An intraluminal drain was always left behind, and was removed at the time of the first bowel movement or on the second postoperative day in case of a protective ileostomy. In the event of a negative air leak test, protective ileostomy was mainly used in case of lower-third tumours.

High ligation of vessels with complete TME was also a fundamental criteria during laparoscopic surgery. Surgeries involved a so-called hybrid technique by removing the specimen *via* a Pfannenstiel incision, with the use of an abdominal wall protector. The head of the circular stapler was sutured in during the open stage. In terms of drainage and protective ileostomy, the same technique was used as in open surgery.

### Side effect profile analysis

One of the most important endpoints of the study was to compare and analyse the side effects of the two neoadjuvant oncological treatments. Rates of diarrhoea, radiocystitis, proctitis, weight loss, hand-foot syndrome, nausea, cytopenia, sepsis, anaemia and special treatment-related side effects were analysed.

Side effects were graded based on the Common Terminology Criteria for Adverse Events [20].

### Surgical result analysis

In addition, effect of the changed neoadjuvant oncological treatment on the outcome of surgical treatment was also assessed. Patient quality of life after the two different approaches of neoadjuvant chemoradiotherapy was analysed. Previous studies showed that there has been considerable increase in the number of organ-preserving procedures during minimally invasive surgery [21‐26].

Change in the ratio of resection/extirpation during laparoscopic surgeries has also been evaluated.

### Assessment of perioperative complications

Results were also compared by neoadjuvant treatment and surgical technique. Rate of anastomotic failure, intestinal passage disorder and other complications, such as wound suppuration, were also analysed.

Anastomotic failure was established after appropriate diagnostic imaging [abdominal ultrasound (US) and/or abdominal CT] were performed for abdominal pain, intestinal paralysis and increasing inflammatory parameters (white blood cell, procalcitonin, C-reactive protein, fever, tachycardia and oliguria) with anastomosis insufficiency confirmed during redo surgery.

Intestinal passage disorder was defined as a lack of bowel movement within seven postoperative days or a lack of stool passing through the protective ileostomy within 96 h after surgery.

### Assessment of pathological parameters

The following data was evaluated: distal and circumferential resection lines distance, and number of removed regional lymph nodes. Identical measures (laparoscopic with laparoscopic; open with open surgery) were compared during both periods. Evaluation of the Mandard score (TRG) was the most important parameter, during the assessment.

(1) TRG analysis: The efficacy of neoadjuvant oncological treatment was confirmed through pathological processing of the specimen obtained during post-treatment surgery. TRG ranges from 1 to 5. (Mandard score 1: best regression; 5: worst regression) [27].
(2) Distal- and circumferential resection margins: Assessed if any difference in distance (mm) from the tumour to the distal/circumferential resection margin between oncological protocols and surgical techniques were observed.
(3) Lymph node status: Both oncological protocols and both types of surgeries were assessed for any difference in number of removed regional lymph nodes.

### Comparison of CT images and pathological regression

Informative value and ability of CT scans performed after neoadjuvant oncological treatment to determine the level of tumour regression was assessed. The analysis involved comparing description from the second CT scan to determine TRG during pathological assessment.

### Follow-up

For patients in the surgical arm, follow-up visits were held 1 week, 1 month and 1 year after surgery with continuous oncological follow-ups [17,28].

### Statistics

Statistical analyses were performed with the STATA 16 program (StataCorp, College Station, Texas 77845 USA). The normality of continuous variables was checked by the Shapiro-Wilk test. Two-sample t-test and one-way ANOVA were used to compare the means of two or more samples, respectively. If the distribution was not the normal distribution, then the Wilcoxon rank sum test or the Kruskal-Wallis test was applied. The proportions were analysed using the Chi-squared test and the Fisher exact test. Henceforward, significant results are indicated using asterisks (*p ≤ 0.05; **p ≤ 0.01; ***p ≤ 0.001). The abbreviation “ns” will be used for non-significant *p* values.

## Results

### Patients–Demographics

During the study, data of 185 patients were evaluated. There were 60 female and 125 male patients (Chi-squared test *p* = 0.43) with mean age of 62.8 years in women and 63.1 years in men (Two-sample student t-test *p* = 0.858).

Both oncological treatments were the same with regard to mean BMI (Body Mass Index) of patients (26.8 ± 5.1 [5-FU] vs. 26.9 ± 5.3 [capecitabine]) (Two-sample student t-test *p* = 0.905).

Most frequent tumour location was in the middle third [48.6% (5-FU) vs. 43.6% (capecitabine)] followed by the lower third [30.6% (5-FU) vs. 30.2% (capecitabine)] of the rectum, and the majority of tumours showed concentric, “napkin ring”-like spreading.

### Diagnostical investigations results

A CT scan was performed in all 185 cases, with radiologically visible lesions in 183 cases (98.9%). A second CT scan was carried out after the completion of treatment in 141 cases (76.2%). MRI scan was conducted in 14.7% of cases during the previous oncological protocol and 29.7% during the modified oncological protocol. A second MRI was performed after neoadjuvant oncological treatment in 5.2% and 21% of cases (5-FU vs. capecitabine). The ratio of cases involving endosonography increased over time. An endoscopic US was conducted before the previous (5-FU) and modified (capecitabine) oncological protocols in 40.4% and 61.8% of cases, respectively.

Radiological imaging confirmed that patients usually had T3 [85.1% (5-FU) vs. 85.7% (capecitabine)] and T4 [13.8% (5-FU) vs. 13.2% (capecitabine)] tumour stage with N0 or N1 lymph node involvement. Only 15 patients with an N2-stage rectal tumour were enrolled in the study (Table 1).

### Side effect profile analysis of oncological treatments

(a) Diarrhoea: a change in bowel habits was a relatively common side effect in both oncological protocols. It occurred in 54.3% and 56.0% with intravenous chemotherapy and oral capecitabine, respectively. The main difference was in the grade of diarrhoea, which was more severe (grade 2–3) with 5-FU (Table 2) (Fisher exact test *p* = 0.0352).
(b) Radiocystitis: urinary tract problems occurred in 16.0% and 27.5% of patients in the 5-FU and capecitabine group, respectively. It should be noted, however, that despite being less frequent, the symptoms in the 5-FU group were with a higher rate of grade 2–3 radiocystitis (Table 2) (Fisher exact test *p* = 0.168).
(c) Proctitis: it was more common in the group taking oral capecitabine [19.8% vs. 9.6% (capecitabine vs. 5-FU)] (Fisher exact test *p* = 0.0493) (Table 2).
(d) Weight loss: patients receiving iv. chemotherapy showed notable weight loss compared to those treated with the oral drug. Whereas significant weight loss only occurred in two cases (2.2%) in the capecitabine group, it was observed in 16 cases (17.0%) of the 5-FU group (Fisher exact test *p* = 0.00067) (Table 2).
(e) Hand-foot syndrome: the considerable prevalence of unplanned hospital admission required hand-foot syndrome previously reported could not be confirmed in the capecitabine group—with only one case confirmed during modified oncological treatment [10,29] (Table 2).
(f) Nausea: The leading symptom of iv. chemotherapies. Nausea and vomiting were also predominant side effects in this study during the previous oncological protocol—occurring in 38.3% in the 5-FU group and in only 15.4% of the capecitabine group (Fisher exact test *p* = 0.00045) (Table 2).
(g) Cytopenia, sepsis: no significant difference was demonstrated between the two oncological protocols in the production of cellular blood components (thrombocytopenia ns. *p* = 0.5143; neutropenia ns. *p* = 0.7384). Cytopenia was slightly more common in the 5-FU group, with two related cases of life-threatening febrile neutropenia (Table 2).
(h) Anaemia: there was a non-significant difference in the rate of treatment-emergent anaemia. Anaemia was observed in 33.0% and 22.0% during pre-treatment with 5-FU and oral capecitabine, respectively (Fisher exact-test ns. *p* = 0.0941) (cut-off values: males: haematocrit: 0.39%; haemoglobin: 133 g/L; females: haematocrit: 0.36%; haemoglobin: 118 g/L) (Table 2).

**TABLE 2:** Occurrence of side effects by oncological protocol.

Types of side effects	5-FU (*n* = 94)	Capecitabine (*n* = 91)	*p*-values of Fisher exact test	Grade 2–3 side effect in 5-FU group	Grade 2–3 side effect in capecitabine group	*p*-values of Fisher exact test
Diarrhoea	51 (54.3%)	51 (56.0%)	*p* = 0.8068	14 (14.9%)	5 (5.5%)	*p* = 0.0352
Radiocystitis	15 (16.0%)	25 (27.7%)	*p* = 0.057	9 (9.6%)	4 (4.4%)	*p* = 0.168
Proctitis	9 (9.6%)	18 (19.8%)	*p* = 0.0493	NA	NA	NA
Weight loss	16 (17.0%)	2 (2.2%)	*p* = 0.00067	NA	NA	NA
Nausea-vomiting	36 (38.3%)	14 (15.4%)	*p* = 0.00045	NA	NA	NA
Hand-foot syndrome	0 (0%)	1 (1.1%)	*p* = 0.3081	NA	NA	NA
Anaemia	31 (33.0%)	20 (22.0%)	*p* = 0.0941	NA	NA	NA
Thrombocytopenia	11 (11.7%)	8 (8.8%)	*p* = 0.5143	NA	NA	NA
Neutropenia	14 (14.9%)	12 (13.2%)	*p* = 0.7384	NA	NA	NA
Febrile neutropenia	2 (2.1%)	0 (0%)	*p* = 0.1617	NA	NA	NA

The following treatment-related complications should be noted: two cases of hepatotoxicity in the 5-FU group and one case of angina in the capecitabine group. The latter warranted a dose reduction in chemotherapy.

### Timing of surgery

During both study periods, patients underwent surgery after the range of 8 up to 10 weeks of completed oncological treatment. During the first period, mean time to surgery was 59 days and 57 days during the period of modified protocol.

### Surgical treatment

Based on our results, 27.9% more laparoscopic interventions were performed after the new, modified neoadjuvant treatment. Organ-preserving surgery was performed in 55.3% of cases after the previous neoadjuvant CRT potentiated with 5-FU, while this ratio was 63.7% in patients treated with the modified oncological protocol. This result did not prove to be significant; however, it may indicate an improving trend in surgical treatment and level of the patient comfort (Fisher exact test *p* = 0.0795).

There was no significant difference in a number of sphincter preserving surgery according to a different neoadjuvant oncological protocol, but due to the laparoscopic surgery, we could perform significantly higher rate of sphincter preserving procedures. The percentage was 72.3% and 39.7% (laparoscopy vs. open surgery (Chi-squared test *p* = 0.00001).

At our department, during the first treatment period (5-FU) the percentage of laparoscopic surgery was 46.8% respectively. During the modified neoadjuvant treatment period (capecitabine), this percentage shifted to 74.7%, respectively, which was significant elevation and proved to be better than results from many international studies (Chi-squared test *p* = 0.0001).

### Perioperative complications

#### Anastomotic failure

Anastomotic failure comprises special importance, not only with regard to different neoadjuvant protocols, but also to both types of surgical interventions. Among patients pre-treated with 5-FU, redo surgery was required in one case (1.06%) after open surgery—deviation, resuturing and lavage/drainage were performed. Redo surgery was also required once (1.09%) after capecitabine treatment in case of laparoscopic surgery, during which Hartmann’s procedure was performed.

#### Intestinal passage disorder

The rate of passage disorder following laparoscopy was 6.4% and 3.3% in the 5-FU and capecitabine groups, respectively. This rate was 8.5% (5-FU) and 8.7% (capecitabine) after open surgery. When only the pre-treatment method is taken into account, regardless of the type of surgery, a passage disorder was diagnosed in 14.9% and 12.1% for 5-FU and capecitabine groups, respectively.

#### Impaired wound healing

Overall, the number of surgical wound infections was relatively low. In case of open surgeries, this occurred in 9.6% (5-FU) vs. 3.3% (capecitabine), while 6.3% (5-FU) vs. 8.7% (capecitabine) in case of minimum sized incisions during laparoscopic surgeries. In case only the pre-treatment method was taken into account, regardless of the type of surgery, wound healing disorder was diagnosed in 16.0% and 12.1% for 5-FU and capecitabine groups, respectively. No redo surgery was required for impaired wound healing, since a few days of local treatment without antibiotics was sufficient in all cases.

### Pathological results

#### Tumour regression grade

Data of 185 patients were classified in concordance with the Mandard score. Complete regression was achieved in 36 cases, of which 23 were observed as a result of the modified neoadjuvant CRT potentiated with capecitabine (Table 3). After aggregating number of cases in TRG2, TRG3, TRG4 and TRG5 groups, the proportion of TRG1 was significantly higher in capecitabine treatment compared to 5-FU treatment (Chi-squared test *p* = 0.049).

**TABLE 3:** Grouping TRG values by oncological protocol.

Tumour regression grade	TRG1	TRG2-TRG5
5-FU (n = 94)	13 (13.8%)	81 (86.2%)
Capecitabine (n = 91)	23 (25.3%)	68 (91%)
*p*-value of Chi-squared test	*p* = 0.049	

TRG1 compared to aggregated number of cases in TRG2-5. The difference was significant with Chi-squared test *p* = 0.049.

#### Distal and circumferential resection margins

After laparoscopy, the distance of distal resection margins were 28 mm (5-FU) vs. 23 mm (capecitabine), respectively (one-way ANOVA test ns. *p* = 0.852). Following open surgery, this distance was 17 mm (5-FU) vs. 14 mm (capecitabine) respectively (one-way ANOVA test *p* = 0.296) (Table 1).

After laparoscopy, the distance of circumferential resection margins were 13 mm (5-FU) vs. 13 mm (capecitabine). Following open surgery, this distance was 9 mm (5-FU) vs. 10 mm (capecitabine), respectively. Based on these results, there was no significant change in the distance from either the distal, or the circumferential resection surface after modifying neoadjuvant treatment. Significant changes were only seen in distal resection margin distance by type of surgery (open vs. laparoscopic, *p* = 0.0077 one-way ANOVA test) (Table 1).

#### The number of removed lymph nodes

The average number of lymph nodes removed during laparoscopic surgery was 8.7 (5-FU) vs. 10.7 (capecitabine), respectively. Hence, it was possible to remove an average of two, or more lymph nodes per surgery during the second study period during laparoscopic surgeries (*p* = 0.0111 Two sample Wilcoxon rank-sum test).

No difference was observed after open surgeries. The average number of removed lymph nodes was 8.0 (5-FU) vs. 8.0 (capecitabine), respectively.

### Comparison of CT images and pathological regression

According to results, tumour response described during the second CT only correlated with TRG in 48.9% (69/141 scans). Based on follow-up CT scans, tumour response to neoadjuvant oncological treatment was classified as better or worse than the result from the postoperative pathological assessment in 12.2% and 38.9% of cases, respectively. These results confirm the well-known fact that CT scans are not appropriate for measuring tumour response after neoadjuvant oncological treatment [30,31].

## Discussion

Successful treatment of rectal tumours requires complex care, the main pillars of which are proper diagnostics, oncological therapy with continuously advancing new drugs and procedures, and properly planned and performed surgical treatment. It is important to support the efficacy of the modified oncological treatments with real-world results.

In accordance with previous protocols, neoadjuvant oncological treatment was mainly initiated for stage T3–4, resectable rectal tumours and less frequently for those of stage T2, at our department. This was due to tumour size, local spreading and/or lymph node involvement and the proximity of the sphincter.

This therapy bears numerous advantages over the adjuvant treatment. It was demonstrated to decrease tumour size (downsizing), hence tumour regression may occur with a favourable response (downstaging). Both downsizing and downstaging contribute to the increased ratio of sphincter preserving procedures, with considerable improvement of patient quality of life. Neoadjuvant treatment of locally advanced rectal cancer (LARC) moreover increases the rate of recurrence-free survival. An argument of preoperative treatment is that pelvic tissues have better blood and oxygen supply before planned surgery, which improves treatment sensitivity. At the same time, ability to regenerate also shows better results before postoperative adjuvant therapy [32].

Effect of neoadjuvant therapy on improved survival has been demonstrated in numerous publications [33–35]. However, besides these advantages, short- and long-term disadvantages of neoadjuvant therapy should also be mentioned. Bin et al. [36] have reported side effects of preoperative neoadjuvant radiotherapy and chemoradiotherapy. According to the meta-analysis of more than 41,000 patients, pre-treatments were associated with a significantly higher number of impaired wound healing. However, these did not affect the incidence of anastomotic failure, and rate of postoperative intestinal obstruction, or passage disorders.

Our study, not only confirmed this observation, but there was no relationship found between the modified pre-treatment and the above mentioned complications.

Postoperative mortality/morbidity-increasing effect of radiotherapy has always been an important factor, as well as weighing acute Grade 3–4 toxicities due to combined CRT in the prevention of local recurrence [36,37].

Neoadjuvant radiotherapy on its own increases the rate of wound healing complications and impaired wound healing potential. This is a well-known phenomenon, since radiation causes damage to DNA and, and the proteins as well. It seems as radiotherapy prompts a considerable increase in cytokine production, thus increasing local inflammation, the accumulation of cell matrix and the severity of developing fibrosis. The resulting drop in matrix metalloproteinase and nitric oxide levels may also be regarded as being responsible for inadequate tissue recovery and regeneration [38,39]. The short-term 25-Gy treatment with previously used immediate surgical intervention, triggers a strong inflammatory response with higher overall morbidity rate. These results have already been clearly demonstrated by the Stockholm III study [40,41]. Our work group thus also chose the long-term treatment with delayed surgical intervention after 8–10 weeks of neodjuvant CRT.

It is important that we did not employ high dose radiation during our treatments, meaning 5 Gy daily, which could have raised the rate of postoperative inflammatory responses, various complications and even sphincter dysfunction. There is now international consensus that the number of expected side effects is considerably lower when a daily dose of 1.8 Gy (also used by us) is delivered [42].

When analysing chemotherapy components of the combined neoadjuvant treatment, a more favourable side effect profile is clearly apparent from present study. There was marked difference in the rate of weight loss compared to previous 5-FU treatment. The shift in the rate of weight loss is also explained by the fact that the severe, Grade 2–3 diarrhoea occurred with significantly higher ratio during 5-FU treatment (Fisher exact test *p* = 0.0352). Therefore, there was a non-significant difference in severity of radiocystitis between the treatment groups (Fisher exact test *p* = 0.168). There were higher rate of grade 2–3 radiocystis in the 5 FU treated group. The main difference in haematopoiesis plays pivotal role, especially when considering the fact that a greater need for transfusion due to an increased rate of anaemia may entail further undesired immunosuppression. The difference of more than 10% in the rate of treatment-emergent anaemia is certainly remarkable (Fisher exact test *p* = 0.0941).

What is more, certain life-threatening side effects—occurring with the 5-FU treatment—were almost completely absent when capecitabine was used; for example, the rate of potentially fatal febrile neutropenia was lower (2.1% vs. 0.0%; 5-FU vs. capecitabine).

During the development of oncological protocols as part of the NSABP R-04 study, patients pre-treated with 5-FU or capecitabine subsequently received oxaliplatin. The study showed that regardless of fluoropyrimidine derivative administered to the patients, the results were similar. For example, the rate of the three-year locoregional control was 11.2% vs. 11.8% (capecitabine vs. 5-FU). Similarly, five-year survival proved to be 80% vs. 81%. The five-year disease-free survival was 66% vs. 68% in the two groups with different pre-treatments, respectively.

Strong results were previously achieved with the addition of levamisole to 5-FU treatment; disease recurrence and mortality were decreased by 41% and 33%, respectively, in patients with stage III disease [43,44]. It was evident that the combined 5-FU + leucovorin treatment caused the one-year survival rate to rise from 43% to 48% with chemotherapy response rate from 12% to 23% compared to previous monotherapy [45].

Interestingly, the addition of oxaliplatin did not lead to higher pCR rates and did not improve survival, locoregional control or the number of sphincter preserving surgeries. However, it did increase the rate of diarrhoea and, more specifically, the rate of more severe, Grade 3–4 cases.

During our study, we not only assessed the effects of the two different neoadjuvant oncological treatments for rectal tumours, but also evaluated the results according to type of surgery, where, aiming at complete homogeneity, results from open surgeries were only compared to those from other open ones, and laparoscopic results were only compared to data from patients subjected to laparoscopy. Complications occurring in the immediate perioperative period, such as ratio of anastomotic failure, was the same in both study periods. In concordance with international standards, this can be explained with the high number of protective ileostomy cases, comprising true protective effect. Loop ileostomy was employed in cases where anastomotic failure was later potentially expected. As for passage disorders and wound suppuration, slightly more favourable results were observed, in cases where capecitabine was added to the treatment; however, these did not reach a level of significance.

Increased efficacy after treatment modification was clearly evident from histological findings. This was likely caused by several factors, one of which is that 5-FU is an active chemotherapeutic agent acting uniformly throughout the body, while capecitabine is converted into the anti-tumour cell agent 5-FU by radiotherapy—by thymidine phosphorylase overexpressed in irradiated tumour cells—and therefore mainly acts “locally” [46].

A more favourable tumour response to oncological treatment was confirmed based on results of the Mandard score. Improved efficiency of the easy-to-dose oral, sensitising chemotherapeutic agent, compared to iv. 5-FU can be accurately measured and easily standardised through TRG. This marked difference reflected by our results, is one of the most important messages of the current study. The rate of complete pathological tumour regression was almost twice as high after capecitabine treatment than after previous intravenous therapy (25.3% vs. 13.8%; capecitabine vs. 5-FU) (Chi-squared test *p* = 0.049).

Interestingly, there was a notable increase in the number of removed regional lymph nodes present following modified neoadjuvant oncological treatment. Since there is inverse proportionality between the effectiveness of neoadjuvant oncological treatment and the number of regional lymph nodes in the specimen, the number of removed lymph nodes could not be explained with the modification of the oncological protocol [47]. Therefore, we assessed the ratio of laparoscopic to open surgeries after both oncological treatments. The number of laparoscopic surgeries significantly increased during the second neoadjuvant treatment period, which could be a likely explanation for varying results.

Based on measurements of resection margin distance, modifying neoadjuvant treatment protocol did not increase oncological radicality. Beyond its biological impact, the change in neoadjuvant CRT also had an indirect effect on surgical treatment. Although this change did not prove to be significant, it contributed considerably to an increased rate (10% <) of sphincter preserving surgeries, which led to substantial improvement in the level of the patient comfort. The explanation is complex because the greater effectiveness—also confirmed by TRG—resulted in smaller tumours, which technically facilitated sphincter preserving surgeries in a higher proportion of cases. At the same time, the increase in the rate of sphincter preservation was also aided by the growing number of laparoscopic surgeries, since the laparoscopic to open surgery ratio gradually climbed even over this five-year period. With regard to surgical treatment of malignant rectal tumours, more than a decade ago high-level (1A) evidence has confirmed laparoscopy-assisted surgery to be the gold standard. Compared to open surgery, minimally invasive procedures are associated with less blood loss and less need for postoperative analgesia, with more rapidly recovering intestinal peristalsis, moreover earlier mobilisation and improved aesthetic results. Length of hospital stay can also be significantly decreased. At our department, during the first treatment period (5-FU) the percentage of laparoscopic surgery was 46.8% respectively. During the modified neoadjuvant treatment period (capecitabine), this percentages shifted to 74.7%, respectively, which proved to be better than results from many international studies [48]. Despite the higher financing and acquisition costs associated with drugs containing capecitabine as an active ingredient, the lower rates of complications and toxic side effects, as well as improved standard of life clearly favour a therapy shift. There is no doubt about short-term benefits, in addition to a more favourable side effect profile and slightly better or at least same perioperative and late postoperative complications, tumours show considerably more favourable response with the modified oncological pre-treatment. Capecitabine is easy to standardise and dose, with a convenient oral intake route. Currently, there is still no reliable data on long-term survival, however collection and analysis of relevant data is ongoing [49].

Our retrospective cohort study with relatively large number of included patients, underline the importance of the modified neoadjuvant therapy in advanced rectal cancer. However the new worldwide accepted trend is the TNT in these cases.

## Conclusion

The new neoadjuvant CRT potentiated with capecitabine represents an easy-to-use oral treatment modality with more favourable side effect profile for advanced-stage rectal tumours. A higher rate of complete tumour regression was achieved after treatment. The modified oncological protocol may play a role on favourable surgical outcome and therefore on patient life quality, since following capecitabine treatment, the number of organ-preserving surgeries increased in our series.

## Data Availability

The raw data supporting the conclusion of this article will be made available by the authors, without undue reservation.

## Abbreviations

5-FU, 5-fluorouracil; BMI, body mass index; CRT, chemoradiotherapy; CT, computed tomography; iv, intravenous; MRI, magnetic resonance imaging; pCR, pathologic complete response; RT, radio-therapy; TME, total mesorectal excision; TNT, total neoadjuvant therapy; TRG, tumour regression grade; US, ultrasound.
